# Supplementary material for: PI3Kγ Mediates Microglial Proliferation and Cell Viability via ROS
Source: Cells. 2021 Sep 24;10(10):2534. doi: 10.3390/cells10102534 (PMC8534080; doi:10.3390/cells10102534)
Supplement: Supplementary file 1 [file cells-10-02534-s001.zip › cells-1325218-supplementary.pdf]

PI3Kγ MEDIATES MICROGLIAL PROLIFERATION VIA ROS

Caroline Schmidt, PhD [1], Nadine Schneble-Löhnert, PhD [1], Trim Lajqi, PhD [2], Reinhard Wetzker, PhD [3], Jörg P. Müller, PhD [1*], Reinhard Bauer, MD, PhD [1*]

Supplementary materials

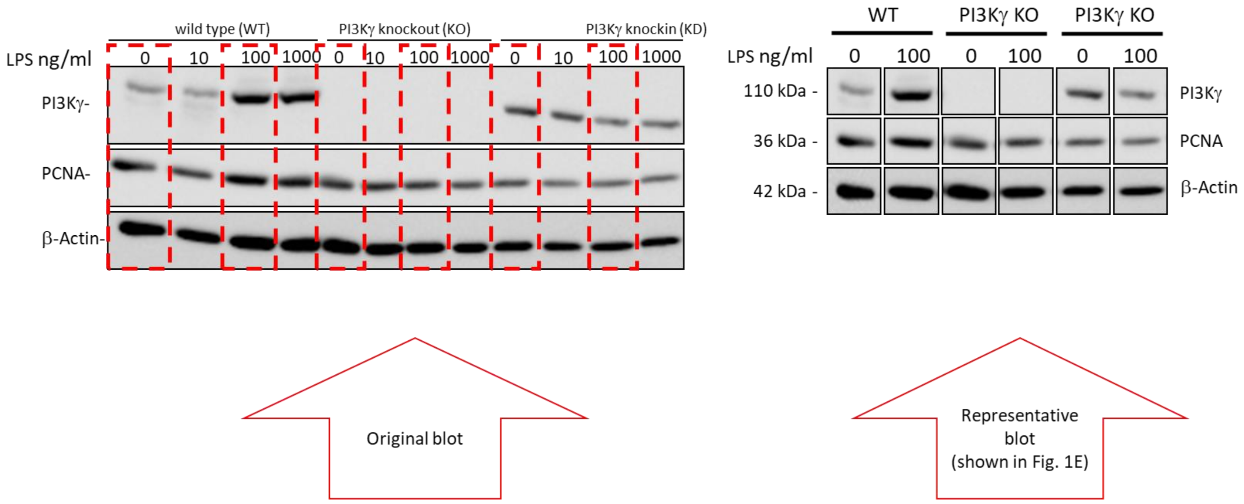

Fig. S1: PI3Kγ controls induction of PCNA in response to LPS mediated stimulation on microglia. Primary microglia derived from wild type (WT), PI3Kγ knockout (KO) or PI3Kγ kinase dead (KD) mice were seeded (100000 cells/well) in a 6-well plate and incubated without LPS: 0 ng/ml and with LPS: 10, 100, 1000 ng/ml. After 6 days cells were harvested, lysed and separated by SDS-PAGE. Subsequent immunoblotting demonstrates cellular level of PI3Kγ, PCNA and loding control ß-actin as indicated.

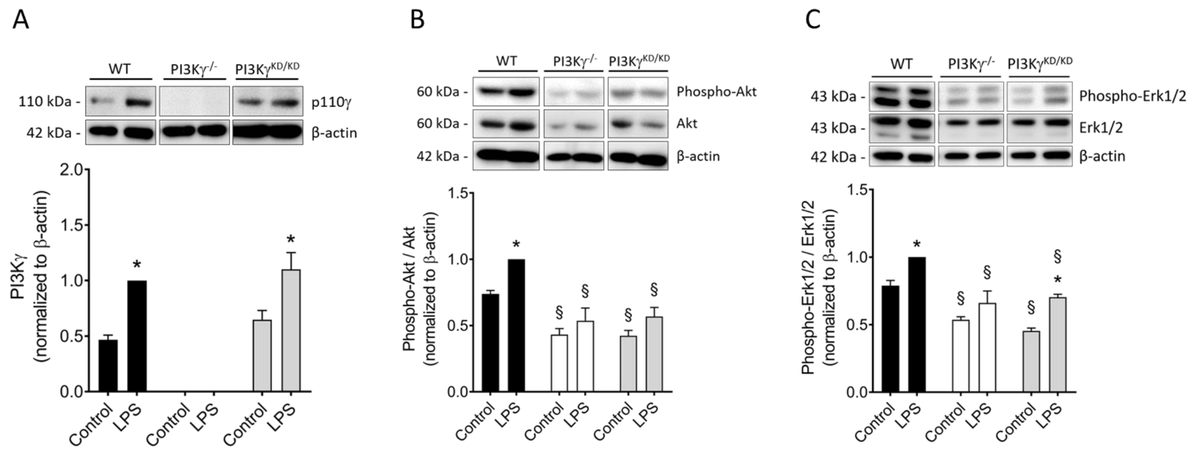

Fig. S2: PI3Kγ mediates Akt and Erk signaling in LPS stimulated microglia. Primary microglial cells isolated from neonatal (wild-type (WT), PI3Kγ KO (PI3Kγ $^{-/-}$), PI3Kγ KO (PI3Kγ $^{KD/KD}$) mice (75000 cells/well) were seeded in 12-well plates on day 0 and were LPS-stimulated (100 ng/ml) on day 6 after seeding. Lysates were collected after 24 h and the protein expressions of PI3Kγ subunit p110γ (left panel), phospho-Akt (middle panel), and phospho-ERK1/2 (right panel) were assayed by Western blotting and quantified (WT LPS-treated cells assigned as 1.0). N = 5, each. $^{*\,§}$ $P < 0.05$, * significant differences vs. control of the same genotype, § significant differences vs. microglia derived from wild type mice at the same condition.

.

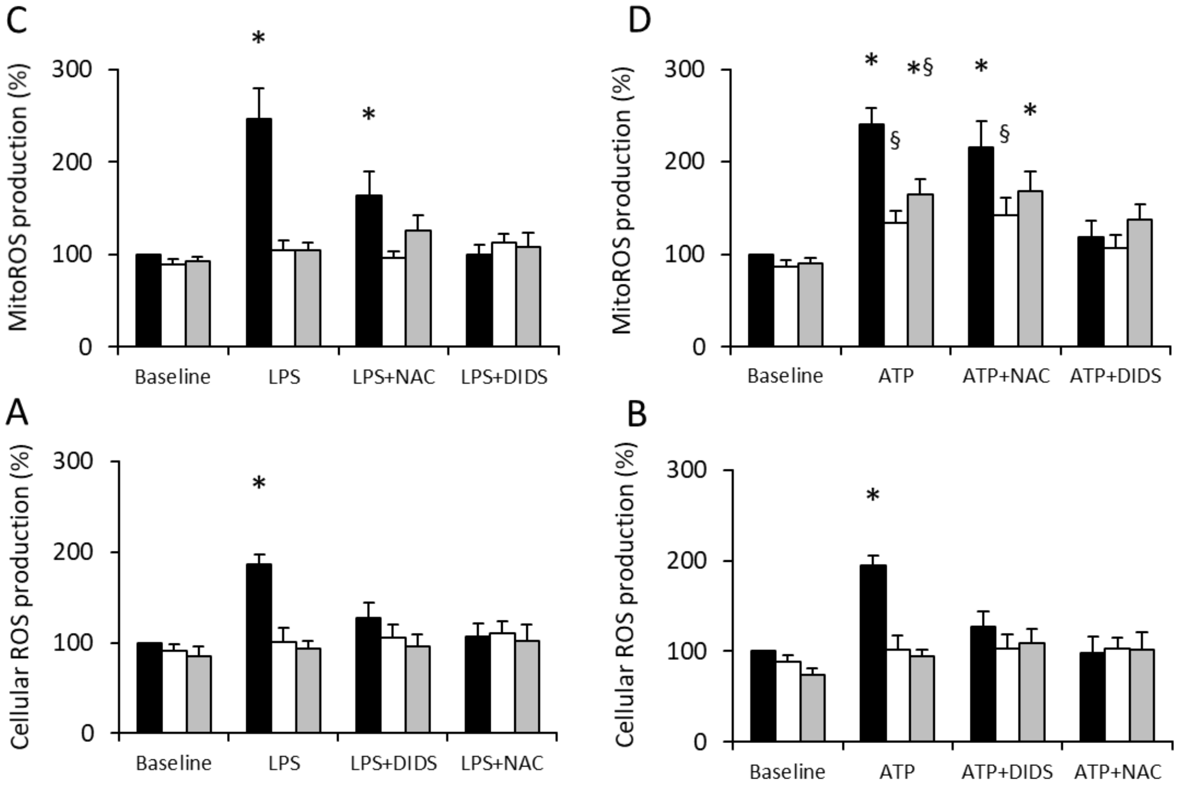

Fig. S3: Inhibitors of cellular and mitochondrial (MitoRos) ROS production abrogate PI3Kγ-mediated increase of ROS production by LPS as well as ATP. Primary microglia derived from wild type (black bars), PI3Kγ knockout mice (white bars), or mice carrying a lipid kinase-dead PI3Kγ mutant (grey bars) were seeded (30000 cells/well) in a 96-well plate and incubated with LPS (100 ng/ml) or ATP (100 µM) solely or together with NAC or DIDS for 6 days. Cellular ROS production was measured by DCF fluorescence. MitoRos production was measured by MitoSOX™ Red mitochondrial superoxide indicator. N = 7-9, each. $*^{§}$ $P < 0.05$, * significant differences vs. wild type baseline, § significant differences vs. microglia derived from wild type mice at the same condition.
